# Supplementary material for: Protocol for developing a Consolidated Checklist for Reporting Mixed Methods Research (CORMIX) using modified Delphi
Source: PLoS One. 2025 May 6;20(5):e0321587. doi: 10.1371/journal.pone.0321587 (PMC12054909; doi:10.1371/journal.pone.0321587)
Supplement: S1 File — (DOCX) [file pone.0321587.s001.docx]

Supplementary file

## Research team:

**Myriam Jaam:**

Myriam Jaam is an accomplished researcher and lecturer at Qatar University's College of Pharmacy with experience in mixed methods research, particularly in the field of medication adherence and diabetes care. Her master's thesis, completed in 2017, titled "Barriers to medication adherence in patients with uncontrolled diabetes in a primary healthcare setting in Qatar: A mixed method triangulation study". This foundational work set the stage for her continued exploration of mixed methodologies. Myriam has since published several papers employing mixed methods approaches. Her expertise in mixed methods is further evidenced by her 2020 SAGE Research Methods Case study, "Investigating medication adherence among patients with diabetes using a mixed-methods approach". This case study demonstrates her ability to not only conduct mixed methods research but also to teach and communicate these complex methodologies to others.

**Ahmed Awaisu:**

Ahmed Awaisu is a distinguished researcher and academic in the field of pharmacy practice and health sciences, with a particular focus on research methodologies including mixed methods and Delphi techniques. He currently holds the position of Professor and Head of Department of Clinical Pharmacy & Practice at the College of Pharmacy, Qatar University. Dr. Awaisu has extensive experience in applying mixed methods approaches to health research. His work frequently combines quantitative and qualitative methodologies to gain comprehensive insights into complex healthcare issues. He has utilized mixed methods in studies on topics such as medication adherence, health-related quality of life, and health services research. He has published over 200 papers in various international journals.

**Derek Stewart**

Derek Stewart is a distinguished researcher and academic in the field of pharmacy practice and health sciences. He currently holds the position of Professor of Clinical Pharmacy and Practice at Qatar University, having previously served as Professor of Pharmacy Practice at Robert Gordon University in Aberdeen, UK. He is the Editor-in-Chief of the International Journal of Clinical Pharmacy. He has published over 250 papers using a range of research methods including mixed-methods. He has particular interest in using theory in intervention development.

Dr. Stewart has also made significant contributions to the development and improvement of reporting tools in healthcare research. Notably, he has been involved in creating extensions to existing reporting guidelines for specific types of studies. For example, he was part of the team that developed the SPIRIT-SURROGATE and CONSORT-SURROGATE extensions for randomized controlled trials with surrogate primary endpoints.

**Banan Mukhalalati**

Dr. Banan Mukhalalati is an Associate Professor at the College of Pharmacy, Qatar University, with a strong focus on health professions education and pharmacy practice research. Her work demonstrates significant expertise in various research methodologies, particularly in qualitative and mixed methods approaches.

Dr. Mukhalalati has made substantial contributions to the field of research methodology in pharmacy and health professions education. She co-authored a chapter titled "Research designs and methodologies related to pharmacy practice" in the Encyclopedia of Pharmacy Practice and Clinical Pharmacy, showcasing her broad knowledge of research approaches in the field. Dr. Mukhalalati has also contributed to the advancement of theoretical frameworks in educational research. Her work on Communities of Practice (CoP) theory, including the development of a theory-informed Communities of Practice Framework for pharmacy and other professional healthcare education programmes, demonstrates her ability to apply and adapt theoretical concepts to educational research.

**Ahsan Sethi**

Dr. Ahsan Sethi is an Associate Professor at QU Health, Qatar University, with a strong background in medical and health professions education. His research profile demonstrates expertise in various research methodologies, particularly qualitative and mixed methods approaches.

Dr. Sethi's methodological expertise is evident in his diverse research output. He has conducted several qualitative studies exploring complex issues in medical education. His expertise in mixed methods research is showcased in studies such as "How do postgraduate qualifications in medical education impact on health professionals?" published in Medical Teacher in 2016. This work combines quantitative and qualitative approaches to provide a comprehensive understanding of the impact of medical education qualifications.

**Marwa Elshazly and Abrar Abdelrahman:**  Both are PharmD students and are working as graduate assistant on this project.

**Muhammad Abdul Hadi**

Dr. Muhammad Abdul Hadi is an Associate Professor of Clinical Pharmacy and Practice at the College of Pharmacy, Qatar University. His research profile demonstrates significant expertise in various research methodologies, particularly in mixed methods approaches and systematic reviews.

Dr. Hadi has made substantial contributions to the field of research methodology in pharmacy practice and health services research. He co-authored a highly cited paper titled "Ensuring rigour and trustworthiness of qualitative research in clinical pharmacy" published in the International Journal of Clinical Pharmacy in 2016. This work has been cited over 850 times, highlighting his expertise in qualitative research methods.

His proficiency in mixed methods research is evident from several publications. Notably, he authored two papers specifically on mixed methods research in pharmacy practice:

1. "Applications of mixed-methods methodology in clinical pharmacy research" (International Journal of Clinical Pharmacy, 2016)

2. "Mixed-methods research in pharmacy practice: basics and beyond (part 1)" (International Journal of Pharmacy Practice, 2013)

These publications demonstrate his deep understanding of integrating qualitative and quantitative approaches in pharmacy research.

Dr. Hadi has extensive experience in conducting systematic reviews and meta-analyses. He has led or contributed to numerous systematic reviews across various topics in pharmacy practice and healthcare. For example:

Dr. Hadi has also contributed to the education of future researchers. He co-authored a chapter on "Mixed methods research in pharmacy practice: Basics and beyond" in the Encyclopedia of Pharmacy Practice and Clinical Pharmacy, indicating his commitment to advancing research methods education in the field.

## Search Strategy

| Database | **Search strategy** | Hits | Search Date |
| --- | --- | --- | --- |
| PubMed | **((((mixed method[Title/Abstract])) OR ((multi method[Title/Abstract]))) OR ((mixed method*[Title/Abstract]))) AND (((((reporting checklist[Title/Abstract]) OR ((reporting standard*[Title/Abstract]))) OR ((reporting guideline*[Title/Abstract]))) OR (reporting tool[Title/Abstract])) OR (appraisal tool[Title/Abstract]))**  ("mixed method"[Title/Abstract] OR "multi method"[Title/Abstract] OR "mixed method*"[Title/Abstract]) AND ("reporting checklist"[Title/Abstract] OR "reporting standard*"[Title/Abstract] OR "reporting guideline*"[Title/Abstract] OR "reporting tool"[Title/Abstract] OR "appraisal tool"[Title/Abstract]) | 1,498 | Sep 5 2024 |
| EMBASE | ('reporting checklist':ab OR 'reporting guideline*':ab OR 'reporting standard*':ab OR 'reporting tool*':ab OR 'appraisal tool*':ab) AND ('mixed method':ab OR 'mixed methods':ab OR 'multi method':ab) | 1,617 | Sep 5 2024 |
| ERIC | AB (mixed method* OR multi method*) AND AB (reporting checklist* OR reporting standard OR reporting guideline OR reporting tool OR appraisal tool) | 34 | Sep 6 2024 |
| CINAHL | AB (mixed method* OR multi method*) AND AB (reporting checklist* OR reporting standard OR reporting guideline OR reporting tool OR appraisal tool) | 946 | Sep 6 2024 |
| ProQuest Central | (abstract(mixed method*) OR abstract(multi method*)) AND (abstract(reporting checklist) OR abstract(reporting standard) OR abstract(reporting guideline) OR abstract(reporting tool) OR abstract(appraisal tool)) | 3,863 | Sep 6 2024 |
| SCOPUS | ( "mixed method" OR "multi method" ) AND ( "reporting checklist" OR "reporting standard" OR "reporting guideline" OR "reporting tool" OR "appraisal tool" ) | 1,734 | Sep 6 2024 |
| ProQuest Thesis and Dissertation | (abstract(mixed method*) OR abstract(multi method*)) AND (abstract(reporting checklist) OR abstract(reporting standard) OR abstract(reporting guideline) OR abstract(reporting tool) OR abstract(appraisal tool))” | 2,115 | Sep 6 2024 |
| PsycInfo | Abstract((mixed method*) OR (multi method*)) AND (abstract((reporting checklist) OR (reporting standard) OR (reporting guideline) | 1632 | Sep 6 2024 |

## ChatBot examples used.

*
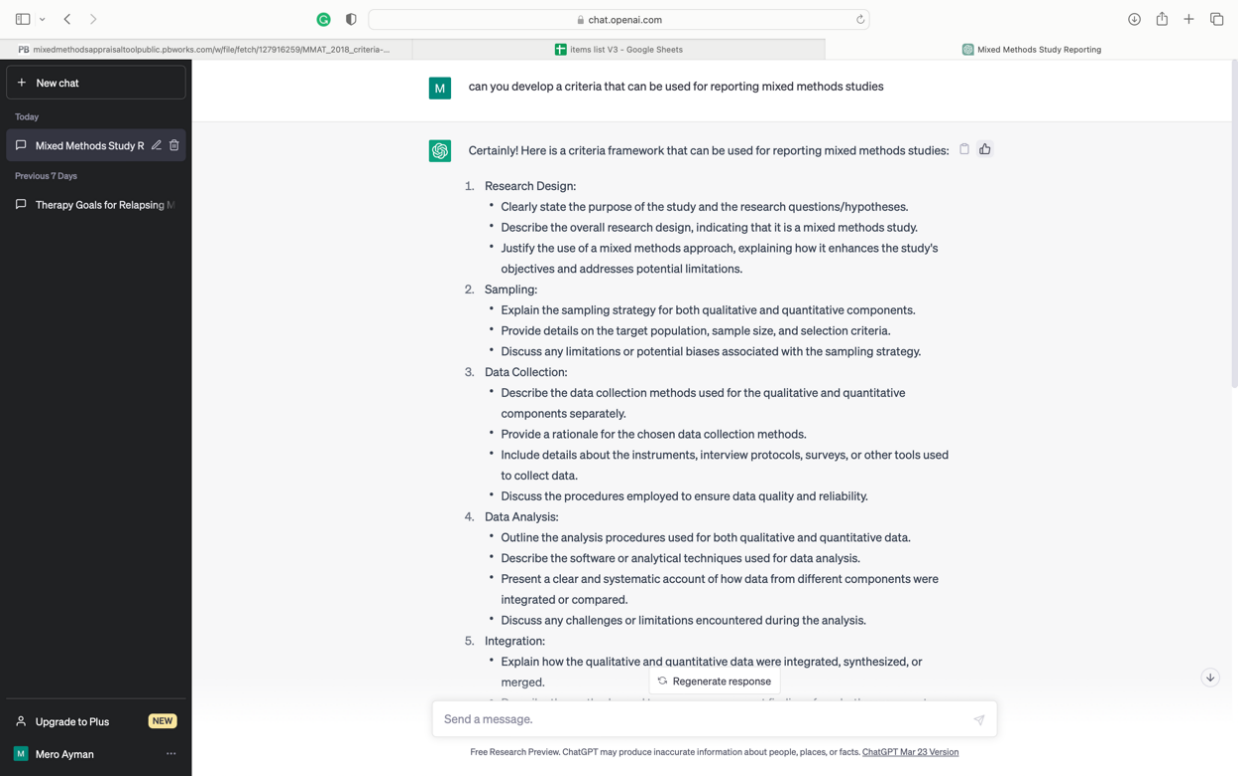

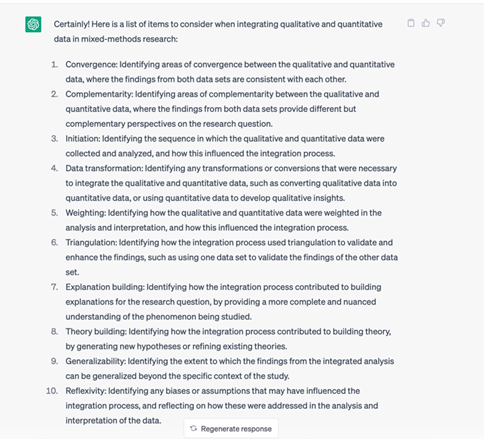
*
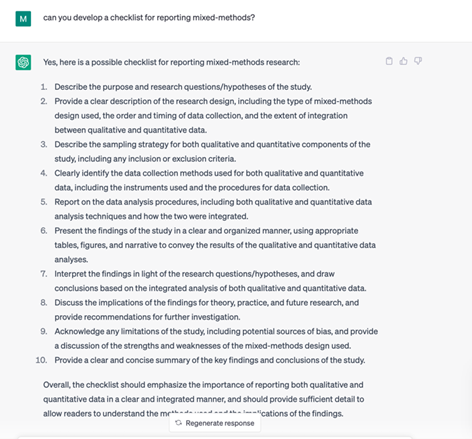
*
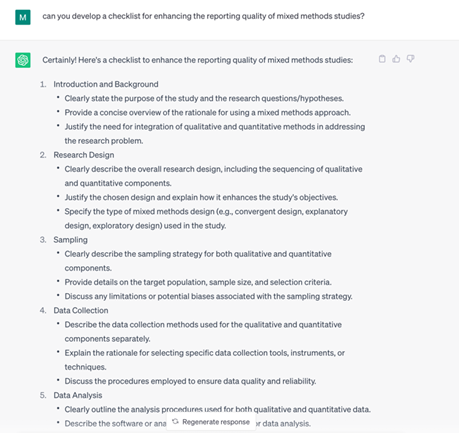
ChatGPT Question 1 and Answer ChatGPT Question 2 and Answer*

*ChatGPT Question 3 and Answer ChatGPT Question 4 and Answer*

## Preliminary items:

| # | Item |
| --- | --- |
|  |  |
|  | 1. Title |
| 1 | Identifies the study as “mixed methods” in the title |
|  | 1. Abstract |
| 2 | ***Type****:* Specify the type of mixed methods research in the abstract (e.g. exploratory, explanatory, sequential, convergent) |
| 3 | ***Information*** ***balance****:* Provide a balanced abstract that encompasses both quantitative and qualitative aspects of the study. |
|  | 1. Introduction/Background |
| 4 | ***Background***: Provide literature on the topic that includes prior quantitative, qualitative, and/or mixed methods research. |
| 5 | ***Context****:* Define mixed methods research within the study context. |
| 6 | ***Rationale****:* Explain the rationale highlighting the knowledge gap on the topic requiring the mixed methods approach. |
| 7 | ***Rationale***: Highlight the study potential significance on the research field. |
| 8 | ***Aim and research question****:* Describe the research aim in a way which clarifies the necessity for using a mixed methods approach |
| 9 | ***Aim and research question****:* State specific objectives for quantitative and qualitative phase of the mixed methods |
| 10 | ***Aim and research question****:* Indicate both primary and secondary (if available) study objectives. |
|  | 1. Research Design |
| 11 | ***Protocol****:* Provide registry or reference to protocol (if available) and highlight if any changes were made to the original protocol with justification |
| 12 | ***Design***: Provide a clear description of mixed methods design used (e.g. convergent, sequential explanatory, sequential exploratory, embedded, transformative, multiphase). |
| 13 | ***Design***: Provide a justification for the use of mixed methods (e.g. triangulation, complementarity, development, initiation, expansion) |
| 14 | ***Design***: Describe the approach to design if fixed design or emergent design |
| 15 | ***Theory/ conceptual framework / paradigm:*** Present the underlying theory or conceptual framework and its relevance to mixed methods research. |
| 16 | ***Theory/ conceptual framework / paradigm:*** Indicate if the study is to test (confirmatory) or develop (exploratory) a theory |
| 17 | ***Theory/ conceptual framework / paradigm:*** Indicate if single or multiple paradigm stance |
| 18 | ***Theory/ conceptual framework / paradigm:*** Indicate what paradigmatic perspective will guide the research design (e.g. postpositivism, constructivism, transformative and pragmatism) |
| 19 | ***Dominance***: Decide on the prominence of each data type in data collection, analysis and results (i.e. whether the study is quantitative-dominant, qualitative dominant, or whether both types are given equal status). |
| 20 | ***Dominance***: Indicate the sequence and timing (i.e. concurrent, or sequential) |
| 21 | ***Dominance***: Identify the level of mixing (i.e fully mixeds [across data collection, analysis, and interpretation] or partially mixeds [only at interpretation stage]) |
| 22 | ***Setting:*** Specify and justify the setting and/or location |
| 23 | ***Ethics***: Discuss ethical considerations related to the study (e.g. anonymity, privacy, confidentiality, consent, incentives) |
| 24 | ***Ethics***: Provide the institution or organization that granted ethics approval for this study or project. |
| 25 | ***Ethics***: Provide the institutional review board approval reference and date |
| 26 | ***Presentation***: Provide a visual representation of the study design and sequence of qualitative and quantitative research components |
|  | 4.a. Quantitative method |
| 27 | ***Design:*** Provide a clear description of the quantitative study design (e.g. observational, interventional) |
| 28 | ***Intervention:*** **Describe the intervention with sufficient details to allow reproducibility |
| 29 | ***Outcome:*** Describe and define primary and secondary outcome measures |
| 30 | ***Data collection:*** Describe the methods and tools used for quantitative data collection and justifying their relevance. |
| 31 | ***Data collection:*** Indicate data collection period |
| 32 | ***Data collection:*** Describe how often data was collected. |
| 33 | ***Data collection:*** Describe personnel involved in data collection |
| 34 | ***Data collection:*** Describe any translation completed |
| 35 | ***Sampling:*** Describe the target population. |
| 36 | ***Sampling:*** Describe the sampling strategy with justification |
| 37 | ***Sampling:*** Describe the sampling frame |
| 38 | ***Sampling:*** Describe the recruitment process |
| 39 | ***Sampling:*** Describe the inclusion and exclusion criteria. |
| 40 | ***Sampling:*** Describe the**group allocation / randomization process including allocation concealment |
| 41 | ***Sampling:*** Describe the**process of blinding |
| 42 | ***Sampling:*** Describe the**number of group(s) |
| 43 | ***Sampling:*** Describe the**control/comparison group(s) |
| 44 | ***Sample size:*** Indicate sample size and its power and precision calculations |
| 45 | ***Analysis:*** Outline the quantitative data analysis process, including the software used |
| 46 | ***Analysis:*** Describe and justify the statistical tests used. |
| 47 | ***Analysis:*** Describe process of addressing missing data and outliers |
| 48 | ***Analysis:*** Describe any subgroup analysis conducted |
| 49 | ***Analysis:*** Describe data transformation |
| 50 | ***Analysis:*** Describe data integration |
|  | 4.b. Qualitative method |
| 51 | ***Design:*** Provide a clear description of the qualitative study design (e.g. Ethnography, phenomenology, grounded theory, case study) |
| 52 | ***Theory:*** Describe the role of the theory in the qualitative phase |
| 53 | ***Data collection:*** Describe the methods and tools used for qualitative data collection and justifying their relevance. |
| 54 | ***Data collection:*** Indicate data collection period |
| 55 | ***Data collection:*** Describe how often data was collected |
| 56 | ***Data collection:*** Describe personnel involved in data collection |
| 57 | ***Data collection:*** Describe any translation completed |
| 58 | ***Sampling:*** Describe the target population. |
| 59 | ***Sampling:*** Describe the sampling strategy with justification |
| 60 | ***Sampling:*** Describe the recruitment process |
| 61 | ***Sampling:*** Describe the inclusion and exclusion criteria. |
| 62 | ***Sample size:*** Indicate sample size and its considerations (e.g. data saturation) |
| 63 | ***Sample size:*** Indicate the number of participants in each data collection session (e.g. In each focus group) |
| 64 | ***Analysis:*** Describe the transcribing process |
| 65 | ***Analysis:*** Name and justify the method of data analysis |
| 66 | ***Analysis:*** Describe the qualitative data analysis process, if inductive or deductive |
| 67 | ***Analysis:*** Describe the coding process, coders involved, and software used |
| 68 | ***Analysis:*** Describe data transformation |
|  | 4.c. Data integration |
| 69 | ***Point interface:*** Describe level of integration (e.g. data collection, analysis, interpretation) |
| 70 | ***Point interface:*** Describe data integration process (e.g. data transformation, following a thread, triangulation) |
| 71 | ***Point interface:*** Use “joint display” tables or graphs to illustrate mixed methods analysis. |
| 72 | ***Point interface:*** Describe software used for data integration |
|  | 4.d. Rigor / Robustness |
| 73 | Describe strategies to enhance credibility, transferability, dependability, reflexivity and confirmability of the qualitative method. |
| 74 | Describe strategies to enhance validity, reliability, replicability, and generalizability of the quantitative phase. |
|  | 1. Results / Findings |
| 75 | ***Alignment:*** Organize results in alignment with the chosen mixed methods design. |
| 76 | ***Participants:*** Describe characteristics of participants in both qualitative and quantitative phase |
| 77 | ***Interpretation:*** Interpret qualitative analysis results with appropriate quotes |
| 78 | ***Interpretation:*** Interpret quantitative analysis results in consideration of statistical and/or practical significance |
| 79 | ***Interpretation:*** Interpret the results of data integration |
| 80 | ***Incomplete*** ***data:*** Address incomplete data (nonresponse, attrition, missing) in interpretation. |
|  | 1. Discussion and conclusion |
| 81 | ***Gained knowledge:*** Discuss the insights gained from using mixed methods and how it enhanced understanding of the research problem. (e.g. corroboration, elaboration, complementary, contradiction) |
| 82 | ***Implication:*** Provide in-depth inferences and potential implications and their significance for practice, education, or policy. |
| 83 | ***Consistency:*** Describe and explain areas of consistency and inconsistency between qualitative and quantitative results. |
| 84 | ***Limitation:*** Address limitations or potential biases the research. |
| 85 | ***Limitation*** Describe any limitation of one method associated with the presence of the other method. |
| 86 | ***Strengths:*** Highlight strengths of the mixed methods and overall research. |
| 87 | ***Future recommendation:*** Present recommendations for future research that culminate in a validation, replication, or extension of the underlying study. |
|  | 1. Stakeholders |
| 88 | ***Expertise:*** Describe the research team expertise in mixed methods and their contribution to the research |
| 89 | ***Conflict of interest:*** Disclose any potential conflicts of interest or researcher biases. |
| 90 | ***Funding:*** Describe the funding source and its influence (if any) on the study |
| 91 | ***Wellbeing:*** Mention steps taken to ensure participant and research team well-being. |
