# Supplementary material for: Protocol for developing a Consolidated Checklist for Reporting Mixed Methods Research (CORMIX) using modified Delphi
Source: PLoS One. 2025 May 6;20(5):e0321587. doi: 10.1371/journal.pone.0321587 (PMC12054909; doi:10.1371/journal.pone.0321587)
Supplement: Attachment — Submitted filename: Reviewer_reports_PONE-D-24-20917R3.pdf [file pone.0321587.s005.pdf]

Reviewer 1

A this point, I still have a number of comments, most of which are rather small.

On lines 117-118, the authors state that their tool will address the “philosophical underpinnings of mixed methods,” and on 178-179 they state the need to prompt “researchers to report their philosophical or paradigmatic stance.” But isn’t this something that the experts should decide, rather than having the authors make prior assumptions about the centrality of this topic?

On line 200 they mention searching in the Journal of Mixed Methods Research, and I would add the International Journal of Multiple Research Approaches.

On line 267 the authors state that they will conduct “a targeted literature search to identify additional mixed methods experts.” I find this to be too vague, since it doesn’t say anything about which literature will be searched or what criteria will be used for selection. I have personally conducted several citation searches that being with “mixed method*” and the results are so widely scattered that they would be useless for locating experts in the field.

On lines 272-273 the authors say they will use snowball sampling to expand their list of experts. This is a useful strategy, but snowball sampling is highly sensitive to the initial set of “seeds” that are used. So, this reinforces the point that I just made above about the importance of an explicit strategy for selecting the first set of experts.

Line 303 states that the items will be evaluated “using a 5-point Likert scale from highly relevant to non-relevant.” In my experience, this will lead to a highly skewed set of results, because very few of the items will be “non-relevant” after the authors’ intense screening process. I suggest that the scale run from “Minor Relevance” to “Highest Relevance” and that the authors use a 10-point rating scale. The usual argument for using 5-point and shorter scales is that it reduces respondent “burden,” but that is not a major issue when working with a highly educated panel who have all volunteered to engage in a rating process.

On line 303 the rating scale is described as “highly relevant to non-relevant,” but on lines 320-321 it is described as “Strongly disagree” to “Strongly Agree.” Again, no one is like to strongly disagree with the relevance of these items. If items “pile up” at the top of the rating scale, then al most all of them will meet the authors’ criteria for consensus, which would lead to a very lengthy instrument. As above, I would recommend a 10-point scale, and then use scores of either 7-10 or 8-10 as a cut-off for consensus.

Line 323 I disagree about whether there is agreement on the figure 85% for consensus. One common joke among Delphi researchers is that “there is no consensus about consensus.” I personally would recommend that 85% be set as an aspirational goal, but that 75% after the second round be considered adequate.

On line 345, I have found that one of the most useful aspects of the comments made by the experts is to adjust the wording of items. Will this be possible?

In the section on lines 347 to 367 it is unclear whether the 10 people are each going to rate all 10 articles. If so, I would prefer to have a sub-sample of the panel rate a larger number of articles. You would have the same number of ratings if five people each rated 20 articles. The reason for this recommendation is that variability among the articles is at least as important as variability among the raters.

Line 360 a random sample of “mixed method studies published in the last three years” will have a population of about 3,000 articles. How will the authors “stratify” such a large population to get just 10 (or 20) matching articles? I also worry that relying on PubMed for half the sample frame will lead to an over-emphasize health-related topics. What about substituting Scopus instead?

Reviewer 2

The authors have addressed the comments of the reviewers. Yet, there are still some remaining issues. The main comment is about the timeline suggested. The authors are planning a search period of the literature until September 2024 (line 225), a recruitment period of experts in September 2024 (line 298), and start the Delphi phase in October 2024 (line 299). Considering that the results of the review will be used to develop the Delphi questionnaire, it does not seem feasible to complete all the steps of the review, and develop and validate the questionnaire within one month. If the authors have already completed the review and questionnaire, they should include the actual date of the search, and state that the review is completed in the protocol.

Other comments are listed below:

- Line 106: replace "GRAMMAS" by "GRAMMS".
- Line 166: Spell out "MMAT" since the first time it appears in the text.
- Lines 182-184: The authors mentioned "The MMR-RHS checklist is tailored for rehabilitation and health sciences. While it may be adaptable to other fields, it might not fully capture the nuances of mixed methods research in other disciplines". To help readers understand, the authors should add an example of nuances of mixed methods not captured in the MMR-RHS.
- Line 219: The authors mentioned using free keywords and controlled vocabulary (MeSH and EMTREE). Yet, it is not clear what controlled vocabulary could be used for this topic. As mentioned previously, if the review is completed, the authors could add the actual search strategy used.
- Lines 238-241: "A two-stage screening process will be employed, first: initial screening will focus on mixed methods-specific guidelines. Second, quantitative and qualitative checklists will be reviewed selectively, prioritizing those frequently cited in mixed methods literature or those that have significantly influenced reporting practices". This description of the screening process is different from what is usually found in reviews (i.e., first, screen titles and abstracts and then full-text papers). The process suggested will involve first identifying guidelines and then checklists. This could be done at the same time (i.e., identifying guidelines and checklist from titles/abstracts and then from full-text papers).
- Lines 241-243: "We will limit our in-depth review to the top 10 most relevant checklists for each methodology (quantitative and qualitative) based on citations." It is not clear why the authors will limit to the top 10 checklists. Also, using number of citations to judge which ones are the "most relevant" can also be misleading. For example, more recent checklists are likely to be less cited but can be more relevant compared to a checklist developed in the 80s.
- Line 282: "This will be supplemented by a targeted literature search to identify additional mixed methods experts." The authors should describe how they will process (e.g., where will they find the literature, how will they select the literature, etc.).
- Lines 381-388: "These studies will be systematically selected to represent a range of disciplines and reporting quality. The ten articles will be identified through a systematic search in PubMed and PsycINFO for mixed method studies published in the last three years. Stratified random sampling of the ten articles will be conducted to ensure representation across disciplines." How will the authors assess the reporting quality of the 10 papers? Also, considering the latter sentence (random sampling), it seems that the authors will first identify all published mixed methods studies from the past 3 years indexed in PubMed and PsycINFO. Then, they will appraise the reporting quality of all identified mixed methods studies and classify them into disciplines. They will then randomly select 10 based on disciplines and reporting quality. This seems to be very time consuming. For example, only in Medline, there are more than 7000 papers published since 2022 that have used mixed methods in their titles.
- Reference #1 refers to two different books by Creswell (Designing and conducting mixed method research VS Research design: Qualitative, quantitative and mixed methods approaches). The authors should check which want they wanted to cite.
- Reference #5 is incomplete. Please check.
- Figure 1: There is a missing word at the end of step 6.
- The authors should avoid using contractions in the manuscript (e.g., there's (line 81), It's (line 118), …)
