# Supplementary material for: Protocol for developing a Consolidated Checklist for Reporting Mixed Methods Research (CORMIX) using modified Delphi
Source: PLoS One. 2025 May 6;20(5):e0321587. doi: 10.1371/journal.pone.0321587 (PMC12054909; doi:10.1371/journal.pone.0321587)
Supplement: Attachment — Submitted filename: Response to Reviewers.docx [file pone.0321587.s003.docx]

Sep 2^rd^, 2024

The Editor-in-Chief

PLOS ONE

Dear Dr. Fàbregues

**Revision of Manuscript**

**PONE-D-24-20917**

**Protocol for Developing a COnsolidated Checklist for Reporting MIXed-Methods Research (CORMIX) Using Modified Delphi.**

The authors of the above-named manuscript would like to thank you for your e-mail dated 24 July 2024, containing the constructive comments for the manuscript. The authors very much appreciate the comments and have revised the manuscript accordingly as attached. We are positive that the comments helped in enhancing the quality and scientific merit of the paper.

Below are separate pages, detailing our responses to the comments of the journal requirements and reviewer.

**Please note that changes are made using “track changes” tool within the revised manuscript.**

Thank you to the editor and the reviewer for the time spent on this manuscript. We look forward to receiving your kind response.

Sincerely yours,

**The Corresponding Author**

**All in all, we thank you for your great review which we believe would add great value to the adjusted manuscript.**

**Detailed Responses to the Comments of the Journal Requirements, Editor and Reviewers**

## Journal requirements:

1. When submitting your revision, we need you to address these additional requirements.

Please ensure that your manuscript meets PLOS ONE's style requirements, including those for file naming. The PLOS ONE style templates can be found at 
<https://journals.plos.org/plosone/s/file?id=wjVg/PLOSOne_formatting_sample_main_body.pdf> and 
<https://journals.plos.org/plosone/s/file?id=ba62/PLOSOne_formatting_sample_title_authors_affiliations.pdf>.

We have addressed the formatting requirements as per the documents provided.

2. Please provide additional details regarding participant consent. In the ethics statement in the Methods and online submission information, please ensure that you have specified (1) whether consent will be informed and (2) what type you obtain (for instance, written or verbal, and if verbal, how it will be documented and witnessed).

We added “Informed written consent will be obtained from experts participating in the Delphi phase through email prior to completing the questionnaire” within the ethical approval section.

3. When completing the data availability statement of the submission form, you indicated that you will make your data available on acceptance. We strongly recommend all authors decide on a data sharing plan before acceptance, as the process can be lengthy and hold up publication timelines. Please note that, though access restrictions are acceptable now, your entire data will need to be made freely accessible if your manuscript is accepted for publication. This policy applies to all data except where public deposition would breach compliance with the protocol approved by your research ethics board. If you are unable to adhere to our open data policy, please kindly revise your statement to explain your reasoning and we will seek the editor's input on an exemption. Please be assured that, once you have provided your new statement, the assessment of your exemption will not hold up the peer review process.

This manuscript presents only as a protocol for the study. No primary data is available at this stage.

**Editor Comments:**

- On page 4, lines 62-66, the authors imply that mixed methods research (MMR) leads to a better understanding of the phenomenon under study and increase the quality of the results. Conversely, mixed methods do not increase the quality of a study but rather allow researchers to answer complex questions, among other things.

We thank the editor for this insightful observation. Upon reflection, we concur with the assessment regarding mixed methods research (MMR). We removed the reference to increasing "the quality of the study" and instead emphasized MMR's capacity to address complex research questions. This modification aligns more closely with the current understanding of MMR's role in research design and methodology.

- Throughout the paper, the authors use the term triangulation as a synonym for integration. The MMR literature describes triangulation as one of the possible rationales for MMR; therefore, using the term to describe integration is inaccurate.

We appreciate the editor's observation regarding our use of terminology. Upon careful review, we acknowledge that our usage of 'triangulation' as a synonym for 'integration' throughout the paper is indeed imprecise. In light of this we revised these terms throughout the paper.

- The authors do not cite the journal article reporting criteria for MMR developed by the American Psychological Association (Levitt et al., 2018). This omission is concerning because these standards fill two important gaps identified by the authors: (1) they were developed by a task force group assembled specifically for this purpose and (2) they provide a detailed and comprehensive set of reporting guidelines for MMR.

We sincerely appreciate the editor bringing this significant oversight to our attention. We acknowledge the importance of Levitt et al.'s (2018) work on Mixed Methods Article Reporting Standards (MMARS) developed by the American Psychological Association task force. We have rectified this omission by incorporating a discussion of MMARS in our manuscript as follows: “The MMARS provides guidelines for reporting mixed methods research in psychology and related fields. While more comprehensive than previous tools, it does not provide the level of detail some researchers might need for comprehensive reporting across all aspects of a mixed methods study. Furthermore, its reliance on cross-referencing with other reporting tools (JARS-Qual and JARS-Quant) can complicate the reporting process, potentially creating challenges for researchers attempting to adhere to a unified set of guidelines. This structure, while comprehensive in theory, may introduce practical difficulties in application, particularly for researchers seeking a more streamlined, self-contained reporting standard for mixed methods research.”

- The Methods section needs to be much more detailed, especially the literature review section, which is weak in terms of reporting. The authors must specify the exact search query that they will use. Additionally, what are the inclusion and exclusion criteria?

We appreciate the editor's feedback regarding the need for more detail in our Methods section. We have taken steps to address these concerns and strengthen our methodology reporting. For the literature review: We acknowledge that our initial description of the literature review process lacked details . To address this, we have expanded this section to include:

a) Search Query: We have added an example search query to illustrate our search strategy. The specific query is (("mixed method*" OR "multi-method") AND (reporting OR checklist OR guideline*)) OR ((quantitative AND qualitative) AND (reporting OR checklist OR guideline*))

b) Inclusion and exclusion criteria: We have explicitly stated our inclusion and exclusion criteria as follows: Articles which discuss reporting criteria or a checklist for mixed- methods research, quantitative, or qualitative research and published in English will be included. Articles that describe how to conduct a mixed methods study or focus on quality assessment criteria will be excluded.

- It is unclear why the terms quantitative and qualitative will be used as separate terms in the search (instead of: (quantitative AND qualitative)), and why articles discussing checklists for quantitative and qualitative research will also be included in the review. There are a large number of checklists in both methodologies and reviewing them all will certainly involve a lot of unnecessary work. Moreover, why review these checklists if the goal is to develop reporting guidelines for MMR?

We recognize the editor's concern about the potential volume of checklists. However, we believe that mixed methods research (MMR) is built upon the integration of quantitative and qualitative approaches, thus by reviewing checklists for these individual methodologies, we gain a comprehensive understanding of the reporting standards that form the foundation of MMR. Insights. By understanding the reporting requirements for individual methodologies, we can ensure that our MMR guidelines are consistent with and complementary to existing standards. To address the issue of overwork, we will implement a two-stage screening process: (1) Initial screening will focus on mixed methods-specific guidelines. (2) Quantitative and qualitative checklists will be reviewed selectively, prioritizing those frequently cited in mixed methods literature or those that have significantly influenced reporting practices.

- As noted by one of the reviewers, the authors seem to confuse reporting quality with methodological quality. For instance, the MMAT is a tool to assess the methodological quality of MMR studies, not the reporting quality, as the authors currently imply.

We sincerely appreciate the editor's observation and the reviewer's insight regarding the distinction between reporting quality and methodological quality. We acknowledge that our manuscript mixed these two concepts in some instances, particularly in our discussion of the Mixed Methods Appraisal Tool (MMAT). To address this important point, we have conducted a thorough review of our manuscript to ensure clear differentiation between reporting quality and methodological quality throughout.

Specifically, we have modified our reference to the MMAT as a quality assessment tool rather than a reporting guideline. For example, we now state: the ‘MMAT, QATSDD, and QuADS primarily emphasize quality assessment rather than comprehensive reporting guidance.'

We have clarified our focus on reporting quality and guidelines, emphasizing that while methodological quality is crucial, our current work specifically addresses the standards and practices for comprehensive reporting of mixed methods research.

- On page 8 the authors describe the weaknesses of existing reporting tools. Specifically, they mention that they are based on the views of the authors, have limited guidance for use, and do not use a robust methodology. These statements should be substantiated. For example, the MMAT is based on a Delphi study with experts (not the views of the authors) and has a detailed manual describing how to use the tool (therefore, it does not have limited guidance for use).

We appreciate the editor's comment regarding our characterization of existing reporting tools. Upon careful review, we acknowledge that our statements were overly broad and did not accurately represent the nuances of all existing tools, particularly the Mixed Methods Appraisal Tool (MMAT).

To address this, we have thoroughly revised the paragraph in question to more accurately reflect the current state of mixed methods research tools, distinguishing between reporting guidelines and quality assessment tools. We also removed generalizations about the weaknesses of existing tools and instead provide a more nuanced discussion of the gaps in the literature specifically related to reporting guidelines for mixed methods research. Additionally, we have clarified that our focus is on developing comprehensive reporting guidelines, rather than quality assessment tools. This distinction is now explicitly stated to avoid any confusion.

- As one of the reviewers argues, what is the function of ChatGPT?

The primary function of ChatGPT in this context is to provide additional perspectives and ideas that may complement the findings from the literature search. The intention is not to rely solely on ChatGPT, but to use it as a brainstorming tool to ensure comprehensiveness and potentially identify novel reporting elements that human researchers might overlook. We reviewed the paragraph to clarify the role of ChatGPT.

- The sampling strategy has several important weaknesses. Selecting the sample from personal contacts and snowball sampling may miss MMR researchers with important expertise on the topic. Instead, a strategy to identify researchers based on a literature search would be much more appropriate and accurate. Furthermore, why should content experts have a rank of associate professor or higher? How will consumer experience with mixed methods be determined? In addition, we know from the literature that researchers conceptualize and operationalize MMR quality differently across disciplines. Furthermore, each discipline has different reporting standards. How will the issue of disciplinary differences be addressed in the sample?

We appreciate the editor's and reviewer’s valuable feedback and have thoroughly revised our approach to ensure a more robust and comprehensive study. Our aim is to produce high-quality work that represents the diverse landscape of mixed methods research. As such, we have implemented the following changes:

Sampling Strategy: We will now use a multi-faceted approach to identify experts:

- Extracting author information from our scoping review
- Conducting a targeted literature search for mixed methods experts
- Contacting mixed methods organizations and prominent journals for expert nominations
- Utilizing snowball sampling as a supplementary method to reach additional experts

Inclusion Criteria: We have removed academic ranking requirements to ensure a more inclusive selection based on expertise rather than hierarchical position. Our revised criteria for experts include:

- Holding a PhD or equivalent degree in any relevant field
- A minimum of 5 years of experience in conducting, publishing, or teaching mixed methods research
- At least three published mixed methods studies or significant contributions to mixed methods literature

Disciplinary Diversity: To address the concern about disciplinary differences, we will:

- Aim for a balanced representation of experts across various fields (e.g., psychology, sociology, health sciences, education)
- During the consensus process, we will explicitly address and document how disciplinary differences in reporting standards are reconciled

Handling Disciplinary Conflicts: In the consensus-building phase, we will:

- Encourage experts to articulate discipline-specific concerns
- Use a modified Delphi technique to address and resolve conflicting viewpoints
- Aim for a set of core reporting standards applicable across disciplines, with the possibility of discipline-specific modules if necessary

Future-Proofing: As recommended by Moher et al., we commit to:

- Regularly reviewing and updating the tool to reflect methodological advancements
- Establishing a mechanism for ongoing feedback from the research community
- Conducting periodic reviews to ensure the tool remains relevant across disciplines

- In conclusion, this protocol has a number of important issues that need to be addressed by the authors. Acceptance of the paper will depend on the authors' ability to address these issues and to make substantial changes in some parts of the study design.

We thank the reviewer for their valuable insight and comments. We hope we have addressed the comments sufficiently and improved the paper clarity.

**Reviewers' comments:**

Reviewer #1: I am of two minds about this article. First, I am not a supporter of checklists in non-quantitative research. Second, if one were to develop such a checklist for mixed methods research, the Delphi procedure that the authors propose would be well-suited to that task.

The origin of my doubts with checklists comes from their use in qualitative research. In particular, the authors refer to SRQR and COREQ, without considering the controversy surrounding such “check the box” ratings of qualitative research (for an early critique, see Barbour, 2001, which has over 4,000 citations in Google Scholar).

The underlying problem is that qualitative methods are so diverse that no one set of criteria can apply to even a majority of the most widely used approaches (e. g., grounded theory, interpretive phenomenological analysis, reflexive thematic analysis, and qualitative content analysis). The broad range of criteria that these methods use to assess rigor is quite different from the far more limited criteria for quantitative methods such as surveys and experiments.

This debate around the use of checklists in qualitative methods poses at least two challenges for these authors: Will they limit their Delphi participants to researchers who agree with their preference for universal standards? And, will they try to apply the same standards to both the qualitative and quantitative methods in their instrument?

We sincerely appreciate the reviewer's thoughtful comments and the important issues raised. We acknowledge the ongoing debate surrounding checklists in qualitative research, as highlighted by the reference to Barbour (2001). Our intention is not to constrain the rich diversity of qualitative methods, but rather to enhance the reporting of mixed methods research. To clarify, our goal is to develop a reporting tool, not a quality assessment tool. We recognize the fundamental differences between qualitative and quantitative research and do not aim to apply uniform standards across these diverse methodologies. Instead, our focus is on improving the reporting of how these methods are integrated in mixed methods research, thus enhancing transparency. The need for such a reporting tool has been identified in the literature, particularly to enhance clarity and reproducibility in mixed methods studies. As the reviewer noted, the Delphi procedure we propose is well-suited for this task, allowing us to draw on diverse expertise to develop a consensual set of reporting guidelines.

Regarding the reviewer's specific questions:

1. We aim to include a diverse panel of experts representing various methodological perspectives, such as mixed methods researchers, academics and journal editors. They will also be of various fields e.g., psychology, sociology, health sciences, education). This diversity will help ensure that our guidelines are flexible enough to accommodate different approaches to mixed methods research. Core elements will be identified through the Delphi process and any discipline specific items will also be clarified in our final report.

2. We will not apply the same standards to qualitative and quantitative methods. Each methodology has its own criteria. Our tool will focus on reporting the integration of these methods in mixed methods research, respecting the unique characteristics of each approach.

As highlighted in the recent editorial from the Journal of Mixed Methods Research that we cited, there is a pressing need for guidance on reporting integration procedures and results in mixed methods studies. Our proposed tool aims to address this gap without imposing rigid quality criteria on individual methodological components.

We appreciate the reviewer's caution against oversimplification, and we will strive to develop guidelines that enhance reporting while respecting methodological diversity.

Moving on to issues related to the authors’ proposed methods, I am quite familiar with Delphi studies, and I found the design and description of the current study to be very thorough, with one small exception. On p. 11, lines 222 -223 there is a reference to having the second round of the expert panel review comments from the first round, but there was no mention of collecting such comments in the first round. I assume this just an omission in describing the first round, since it is traditional in a Delphi to collect such comments along with the numeric ratings.

We greatly appreciate the reviewer’s keen attention to detail regarding our Delphi study design. The reviewer is correct in identifying an unintentional omission in our description of the first round of the expert panel review. This was an unintentional omission. In each round of the Delphi a comment box will be included following each item rating to allow the expert to post any comments they have with regards to the item. We have amended the manuscript to clarify this.

In contrast, I was less satisfied with the section on Finalizing the instrument (p. 13), which relies on a sample of five articles from “different fields.” First, five seems like a very low number, given that the authors expect their checklist to have widespread utilization. Second, I would prefer to see some systematic procedure for selecting those articles.

We appreciate the reviewer’s comment. We have amended this section to provide more details regarding the finalization procedure and increased the number of articles to ten. The updated section now reads as follows “This piloting team will independently apply the checklist to a sample of 5 10 published mixed methods studies across different fields. These studies will be systematically selected to represent a range of disciplines and reporting quality. The ten articles will be identified through a systematic search in PubMed and PsycINFO for mixed method studies published in the last three years. Stratified random sampling of the ten articles will be conducted to ensure representation across disciplines. Feedback will be collected quantitatively using a structured questionnaire used after applying the checklist to each study. The questionnaire will include rating item clarity, relevance, and ease of use on a five point Likert scale. Qualitative comments will also be collected using open ended format question where the rater will document specific issues, limitations, or suggestions for each checklist item and the overall tool” This provides more details on the finalization of the tool.

Overall, the nature of this article as a protocol leads me to emphasize the strengths of the proposed methods for meeting the authors’ stated goals. Still, I think the authors should be more reflexive in their thinking about whether a set of check-the-box criteria are indeed adequate for such a complex task as assessing reporting quality in mixed methods research.
Reference
Barbour, R.S. (2001). Check lists for improving rigour in qualitative research: A case of the tail wagging the dog? BMJ, 232:1115-1117.

We sincerely appreciate the reviewer's insightful comment regarding the complexity of assessing reporting in mixed methods research (MMR) and the potential limitations of a checklist approach.

We acknowledge that MMR, by its nature, involves intricate integration of diverse methodological approaches, making comprehensive quality assessment a complex task. We emphasize that our aim is to develop a tool for reporting rather than methodology evaluation. While we believe that a structured checklist can provide a valuable foundation for assessing reporting, we recognize that it may not capture all nuances of well-reported MMR.

In light of this feedback, we will add a section discussing the potential limitations of a checklist approach and acknowledging the complex nature of MMR reporting in the E&E document and the final report of the CORMIX tool. In terms of the methodology, we will include open-ended questions in our Delphi study to capture experts' views on aspects of MMR reporting that may not be easily quantifiable.

Reviewer #2: General Comments/Summary of the paper
This manuscript presents a protocol for the development of a reporting guideline for mixed methods research. The guideline that will be developed from this project can be relevant for those interested in mixed methods research. The main comments concern the confusion between reporting and methodological quality and the lack of information in the methods section. The comments are detailed below.

Main comments

1. The authors use the terms “mixed methods”, “mixed-method” and “mixed-methods” interchangeably throughout the manuscript (text, table and figure). Textbooks usually use “mixed methods” without the hyphen and with an S at the end of method.

We appreciate the reviewer’s observation regarding terminology inconsistency. In response, we have thoroughly reviewed the entire manuscript and standardized the term to 'mixed methods' throughout.

2. There is a confusion between reporting and methodological quality tools in the introduction (page 5) and initial phase (page 8). For example, they cited the MMAT, QATSDD and QuADS as existing tools that they found (line 137). Yet, these tools were not developed as reporting guideline but for appraising the methodological quality of studies. Reporting guidelines will usually include more criteria to ensure that the information is complete and clear. There are more than 600 reporting guidelines on the EQUATOR Network. More than a dozen mixed methods reporting tools can be found from the EQUATOR Network such as the MMR-RHS checklist and ASSESS tool. The authors should focus on these tools to justify their project and develop their questionnaire. There are also other reporting guidances for mixed methods that the authors can rely on such as Brown, K., Elliott, S., Leatherdale, S., & Robertson-Wilson, J. (2015). Searching for rigour in the reporting of mixed methods population health research: a methodological review. Health Education Research, 30(6), 811-839; Leech, N., & Onwuegbuzie, A. (2010). Guidelines for conducting and reporting mixed research in the field of counseling and beyond. Journal of Counseling and Development, 88(1), 61-70; Burrows, Timothy J. 2013. A preliminary rubric design to evaluate mixed methods research. Ph.D. diss., Virginia Polytechnic Institute and State University, <https://www.proquest.com/dissertations-theses/preliminary-rubric-design-evaluate-mixed-methods/docview/1513244026/se-2>. The authors should consider reviewing this sentence “…these tools use different sets of criteria based on author’s views and experts opinions rather than robust methodology. Standardized scoring system is lacking, and subjective judgement is made by accessors, hence, inter-rater reliability may be inconsistent. (lines 141-143)”. Some tools have been developed using a rigorous process (including Delphi and qualitative interviews). Also, inter-rater reliability is not necessarily a recommended step for the development of reporting guideline (Moher et al., 2010).

We sincerely appreciate the reviewer's thorough feedback and the valuable references provided. We acknowledge the initial confusion in our manuscript between reporting guidelines and methodological quality assessment tools. We have revised our paper to clearly differentiate between reporting guidelines and methodological quality assessment tools. We emphasize on our focus on developing a reporting guideline. We also acknowlede that tools like MMAT, QATSDD, and QuADS are designed for methodological quality appraisal rather than reporting guidance.

We have expanded on MMR reporting guidelines, including those available on the EQUATOR Network. Specifically:

a) ASSESS tool: While comprehensive, we noted its implementation-focused approach and limitations in addressing broader MMR studies. We've incorporated a discussion of its strengths and limitations in our review.

b) MMR-RHS checklist: We've included this in our paper, noting its focus on rehabilitation and health sciences and discussing its potential adaptability to other fields.

c) We've added references to the works by Brown et al. (2015). This review emphasizes the need for a reporting guideline and does not present as a tool on its own, but rather reviewed existing mixed methods studies using the GRAMMAS tool. We have added this reference to support the need for a reporting tool.

d) As for the references by Leech & Onwuegbuzie (2010), and Burrows (2013) they provide a guidance and recommendations on how to conduct and report mixed methods and focus on methodological quality rather than reporting, respectively. We will use insights from all these sources to inform the development of our initial questionnaire for the Delphi study, ensuring a comprehensive foundation for our work.

Additionally, we have revised our statement about the development of existing tools, acknowledging that some have been developed using rigorous processes. We've removed the reference to inter-rater reliability as a necessary step in developing reporting guidelines, aligning with Moher et al. (2010).

3. More information on the research team is needed (page 8). How many persons are part of the team? Are they junior/senior researchers or trainees? What is the expertise of each member? Do they all have expertise/experience in mixed methods? Based on the reference list, it seems that only the last author has expertise in mixed methods.

We added a supplementary file describing the expertise of the research team.

4. In figure 1 (page 7), there is a step (“Step 6: Conduct a face-to-face meeting with the research team and all experts to finalise the CORMIX tool”) that is not described in the text. More information is needed on how this step will be conducted. Who are “all the experts” (e.g., those that participated in the Delphi?)?

we thank the reviewer for bringing this to our attention. We have clarified that the 'face-to-face meeting' refers to an in-person gathering of the core research team and explicitly stated that the experts who participated in the Delphi phase will be invited to join this meeting virtually.

5. Initial phase (page 9) : 1) 7 databases are listed on line 152. Yet, ProQuest is a platform that provides access to several databases. The authors should indicate which databases from ProQuest have been used;

We have clarified the specific databases within ProQuest that will be included in our search. Our text now reads “A comprehensive search will be conducted by two researchers through PubMed, EMBASE, PsycINFO, ERIC, CINAHL, ProQuest (Default selected databses: APA PsycArticles; Australia & NewZealand database; Consumer Health Database; Continental Europe Database; East & South Asia Database; East, Europe & Central Europe Database; Health & Medical Collection; Healthcare Administration Database; Library Science Database; Nursing & Allied Health Database; Psychology Database; Research Library; Social Science Database; Sociology Database; ProQuest One Academic)”

2) The search strategy is not detailed. Did they consulted a specialized librarian to develop the search strategy? Did they used Boolean, truncation or proximity operators? What time frame was covered in the search strategy?;

We thank the reviewer for their feedback. We provided more details within the section describing the scoping review.

3) The authors mentioned searching in the grey literature (line 153). Is the search of the grey literature limited to Google Scholar? If no, please add the other sources used. Also, the “major journals” searched should be listed (line 153);

Regarding the grey literature search, we have clarified that it is specifically limited to two sources: Google Scholar and ProQuest Dissertations & Theses Global. Concerning the major journals, we have added a list of the specific mixed methods research journals that will be searched.

4) The authors mentioned that the data will be extracted using a “Standardised data extraction sheet” (line 161). More information on what data will be extracted is needed;

We appreciate the reviewer's request for more detailed information regarding our data extraction process. We acknowledge that our initial description of the 'Standardised data extraction sheet' was insufficient, and we have expanded this section in our revised manuscript.

5) The authors mentioned, “The use of AI to generate item Chatbot, equipped with language processing, can develop items that can be beneficial for CORMIX. Items generated by ChatBot will be compared with items found from the literature search to supplement the findings. To ensure comprehensiveness, the prompt questions will be repeated in different timestamps and contexts.”(lines 164-167). This method is innovative but would benefit from more precision. For example, what will be asked to generate the items? Is this process replicable by other researchers (e.g., if another researcher use ChatBot with the same questions, will the same items be generated?). Also, what is meant by “repeated in different timestamps and contexts”?

We sincerely appreciate the reviewer's interest in our innovative approach of using AI to supplement our item generation process. We provided more details and clarifications in the manuscript. Different timestamps: Asking the AI the same questions at various times to capture potential variations in responses due to model updates or dynamic knowledge. Different contexts: Rephrasing questions or providing varied background information to elicit diverse perspectives and more comprehensive answers from the AI.

6. Modified Delphi phase (page 10): 1) More information on how the experts will be identified and recruited is needed;

Sampling Strategy: We will now use a multi-faceted approach to identify experts:

- Extracting author information from our scoping review
- Conducting a targeted literature search for mixed methods experts
- Contacting mixed methods organizations and prominent journals for expert nominations
- Utilizing snowball sampling as a supplementary method to reach additional experts

Recruitment will be via email.

2) The authors mentioned aiming for a minimum of 20 experts. What is the rationale behind this number? Is this number after the 1st round or after three rounds? They mentioned that more than 20 (line 181). How many more?;

We have clarified the number of experts needed and we increased the number based on the editor and reviewers comments to 50 experts. This is to capture the diverse disciplines needed. Literature does not provide a specific recommendation with regards to the number of experts in the Delphi study. Yet, we aim to maintain a response rate of at least 75% by the end of the Delphi phase (round 3)

3) The recruitment is expected to start in July 2024 and data collection in August 2024. Thus, the questionnaire and phase 1 is completed. The authors could provide more detailed information on the Initial phase and questionnaire development;

Due to unexpected delays and the summer period; we did not yet start the recruitment process. We amended the dates in the manuscript.

4) The authors mentioned that “(6) An online meeting will be held with the experts between the Delphi rounds to share the main results” (lines 188-189). This is not a typical step in a Delphi method. The results of a round are usually shared at the beginning of the questionnaire of the next round. Could this online meeting influence anonymity (a characteristic of a Delphi method)?;

We are referring to individualized short meetings to reduce dropouts. Only one meeting will be held with all experts to finalize the CORMIX items. We clarified this in the manuscript.

5) It is mentioned that the research team will perform a face and content validation of the questionnaire (line 197). How many persons will rate each item? What are their expertises?

We provided more details regarding the validation. The text now reads “The face and content validation of the questionnaire will be conducted by a panel consisting of the research team members and three external experts. The research team includes five members with expertise in mixed methods research, research methodology, and questionnaire design. The three external experts, identified through professional networks, have extensive experience in conducting Delphi studies. In total, eight individuals will be involved in this validation process.”

7. Finalizing the CORMIX Phase (page 13): How will the raters (2 PhD students and 5 mixed methods researchers) be recruited? How will the 5 published mixed methods studies identified? Will they choose good and bad reported papers? How will the issues or limitations identified from the raters be collected (e.g., using a pre-developed questionnaire vs during an interview or focus group)?

We thank the reviewer for their comment. We provided more information about the finalization stage clarifying the process with more details. We also increased the number of raters to a total of 10 (2 PhD students and eight researchers). The new section now reads “Following the Delphi phase, a meeting will be set up as face-to-face for the research team while Delphi panel experts will be asked to join virtually to go over the CORMIX items for finalization. In this meeting, the final items will be shared, along with the analysis process. Upon confirmation, the checklist will be piloted by a team of two PhD students and eight mixed methods researchers. The PhD students will be recruited from our institution's research methodology program, while the eight researchers will be identified through a combination of purposive sampling from our professional networks and open calls in relevant research forums. All piloting members will have extensive training and expertise in conducting and publishing mixed methods studies. This piloting team will independently apply the checklist to a sample of 10 published mixed methods studies across different fields. These studies will be systematically selected to represent a range of disciplines and reporting quality. The ten articles will be identified through a systematic search in PubMed and PsycINFO for mixed method studies published in the last three years. Stratified random sampling of the ten articles will be conducted to ensure representation across disciplines. Feedback will be collected quantitatively using a structured questionnaire used after applying the checklist to each study. The questionnaire will include rating item clarity, relevance, and ease of use on a five point Likert scale. Qualitative comments will also be collected using open ended format question where the rater will document specific issues, limitations, or suggestions for each checklist item and the overall tool. The pilot team will then convene to consolidate their feedback and any recommended modifications to the checklist. This input will be used in revising and finalizing the checklist”

8. Guidance statement: It is mentioned that the guidance statement “will undergo pilot testing and feedback from intended end-users” (lines 248-249). More information on how this testing is needed (how will they be recruited, contacted, how many, how data will be collected, …).

We appreciate the reviewer’s comment. We provided more information about the guidance statement in the manuscript.

9. Explanation and Elaboration (E&E) Document: Will the E&E also be submitted to testing and feedback as the guidance statement?

The E&E document presents as a transparency documentation of the entire process of developing the CORMIX tool. We aim to publish this document in a peer reviewed journal where it will undergo review for publication.

10. The authors should be careful on their use of the word “triangulation” (lines 66, 76, 147). Triangulation refers of the use of multiple methods or sources of data. In the context of their sentences, they seem to refer more to integration than triangulation. For example, at this sentence, “These reported limitations highlight that researchers merely use mixed methods as a means to collect large volume of qualitative and quantitative data with limited triangulation.”, the word triangulation could be replaced by “integration”.

We appreciate the reviewer’s comment regarding our use of terminology. We revised the terms used throughout the paper.

Minor comments
Line 80: write Equator with capital letters.
Line 112: replace EQUATER by EQUATOR
Line 118: remove “1.” at the end of the sentence.
Line 281: Reference #5 is incomplete.

All comments addressed in the manuscript.

Reviewer #3: More information is needed about the initial phase to clarify the process for the reader, including what the Rayyan tool is, how using ChatGPT benefits the project at this phase, and what you will do with the ChatGPT results.

We thank the reviewer for their comment and review. We added more information with regards to the scoping review clarifying what Rayyan tool is and the role of ChatGPT in the manuscript.

Expert selection: The expert selection process is a bit confusing. First, just because someone is a journal editor of a journal that publishes mixed methods research does not mean that they are an expert in mixed methods quality (unless you are only recruiting from methodological journals such as JMMR, but that does not appear to be the case). Additionally, requiring that content experts/academics be at the rank of associate professor or higher may be limiting. In some fields, mixed methods research is relatively new and assistant professors may know more about it than full professors. For the consumers, how will you know they have a minimum of 3 years of experience in using and publishing mixed-method research?

We carefully revied the experts selection section of the manuscript to address the comments raised by the editor and the reviewers.
