# Supplementary material for: Protocol for developing a Consolidated Checklist for Reporting Mixed Methods Research (CORMIX) using modified Delphi
Source: PLoS One. 2025 May 6;20(5):e0321587. doi: 10.1371/journal.pone.0321587 (PMC12054909; doi:10.1371/journal.pone.0321587)
Supplement: Attachment — Submitted filename: Response to Reviewers R2.docx [file pone.0321587.s004.docx]

Nov 30^th^, 2024

The Editor-in-Chief

PLOS ONE

Dear Dr. Fàbregues

**Revision of Manuscript**

**PONE-D-24-20917R1**

**Protocol for Developing a COnsolidated Checklist for Reporting MIXed-Methods Research (CORMIX) Using Modified Delphi.**

The authors of the above-named manuscript would like to thank you for your e-mail dated 17 Oct 2024, containing the second review with constructive comments for the manuscript. The authors very much appreciate the comments and have revised the manuscript accordingly as attached. We are positive that the comments helped in enhancing the quality and scientific merit of the paper.

Below are separate pages, detailing our responses to the comments of the journal requirements and reviewer.

**Please note that changes are made using “track changes” tool within the revised manuscript.**

Thank you to the editor and the reviewer for the time spent on this manuscript. We look forward to receiving your kind response.

Sincerely yours,

**The Corresponding Author**

**All in all, we thank you for your great review which we believe would add great value to the adjusted manuscript.**

**Detailed Responses to the Comments of the Journal Requirements, Editor and Reviewers**

**Comments from the Editor:**
- Provide a rationale for the feasibility of the review and questionnaire development according to the stated timetable and clarify whether these stages have been completed.

We first thank the editor for their valuable comments and review of this manuscript. We have already completed several key phases of the project. The scoping review has been completed, yielding 50 relevant articles from an initial screening of 4,978 articles. From these articles, we have completed data extraction and synthesis, resulting in a preliminary list of items for the Delphi phase (provided in supplementary file). Additionally, the questionnaire for the Delphi phase has been developed and validated by the research team. Expert recruitment is currently ongoing, and based on our progress and existing commitments from experts, we anticipate completing expert recruitment by end of November, with the first Delphi round beginning in December 2024, followed by subsequent rounds through February-March 2025. This timeline is feasible given the substantial groundwork already completed and allows adequate time for expert engagement between rounds.

- On line 94 you say: “Attempts have been made to formulate reporting guidelines for mixed methods research to improve quality, but comprehensive standards are still lacking”. What do you mean by comprehensive? Please explain further why the published reporting frameworks are not comprehensive.

Thank you for your thoughtful comment. We acknowledge this statement was imprecise and have revised it for clarity. Rather than suggesting existing guidelines lack comprehensiveness, our intent was to highlight the need for a unified reporting tool that addresses both comprehensiveness and practical implementation challenges. As shown in our manuscript, existing tools like GRAMMS and MMARS have made valuable contributions but present specific practical limitations. For instance, MMARS requires users to cross-reference between multiple documents (JARS-Qual and JARS-Quant), which can complicate the reporting process and potentially compromise adherence to the tool. Similarly, while MMR-RHS provides robust guidance, its focus on rehabilitation and health sciences may not fully capture reporting needs across different disciplines. We have revised the text to better reflect these nuances, now reading: "While robust guidelines exist for mixed methods research reporting, there remains a need for a unified, standalone checklist that streamlines the reporting process and provides detailed operational guidance across disciplines."

- On line 106, when discussing the JARS, you state: “While more comprehensive than GRAMMAS, it does not provide the level of detail some researchers might need for comprehensive reporting across all aspects of a mixed methods study”. Please explain this statement further, as we believe that the JARS is quite detailed.

We appreciate this comment and agree that our original statement didn't accurately reflect JARS's detailed nature. JARS-MMARS indeed provides comprehensive guidance for reporting mixed methods research. Our concern was not with its level of detail but rather with practical implementation challenges. We have revised this section to better reflect this distinction. The revised text now reads: "While more comprehensive than GRAMMS, it was primarily developed for psychology and related fields, while mixed methods research spans multiple disciplines including healthcare, education, and social sciences. Additionally, users must cross-reference between multiple documents (JARS-Qual and JARS-Quant), which can complicate the reporting process and compromise adhering to the tool. A standalone, unified checklist could enhance usability and hence, improve reporting quality."

- On line 124, you state that the tool will address “the philosophical underpinnings of mixed methods”. What are these underpinnings? How will they be addressed?

Thank you for this important question. We acknowledge that our statement about "philosophical underpinnings of mixed methods" needs clarification. We have revised this section to be more precise about our approach. Rather than presuming specific philosophical underpinnings that must be reported, our tool will be guided by expert consensus on what aspects of philosophical and paradigmatic considerations, if any, should be reported in mixed methods research. Through our completed literature review, we found that philosophical and paradigmatic considerations are frequently discussed in mixed methods literature. However, we recognize that the decision about whether and how these should be incorporated into reporting guidelines should emerge from expert consensus during our Delphi process rather than being predetermined. Therefore, the role and importance of reporting such considerations will be evaluated through expert consensus in our study. We have amended the manuscript to reflect this more accurately, removing any implications that we have predetermined what philosophical elements should be included in the reporting guidelines.

- Include the full search strategy used or planned as a supplementary file.

We included the full search strategy in the supplementary file.

- Clarify the issues raised by reviewer 2 regarding the screening process and the selection of checklists for review.

We appreciate the reviewer's comment about the screening process and checklist selection. We have revised the manuscript to clarify both aspects. The screening process followed standard systematic review procedures: first, titles and abstracts were screened by two independent researchers using Rayyan, followed by full-text screening of potentially relevant articles. Our search identified 13,439 articles, which after duplicate removal resulted in 4,978 articles for title/abstract screening. Full-text screening was completed for 121 articles, with 50 articles ultimately included.

Regarding the selection of quantitative and qualitative checklists, this was conducted as a supplementary search to complement our primary focus on mixed methods-specific reporting guidance. Any potential limitations from focusing on frequently cited checklists were mitigated through our comprehensive methodology, which includes expert input through the Delphi process to ensure no crucial reporting elements are overlooked.

- If the review has been completed, please indicate the implications of not following the reviewers’ suggestions and how these issues will be addressed. We believe that the reviewers’ comments are important enough to be considered.

We thank the editor and reviewers for their valuable feedback. Although the review has been completed, we have carefully considered the reviewers' comments and addressed them in several ways: First, we have clarified our screening methodology in the manuscript to accurately reflect the systematic process we followed: initial title/abstract screening of 4,978 articles by two independent researchers, followed by full-text review of 121 articles, resulting in 50 included articles. This aligns with standard systematic review procedures. Regarding the selection of quantitative and qualitative checklists, while we initially used citation counts as one selection criterion, we recognize this could potentially miss relevant newer guidelines. However, our methodology incorporates several safeguards against overlooking important reporting elements: the comprehensive capture of items from mixed methods literature, the expertise of our Delphi panel, and the opportunity for experts to suggest additional items or sources during the Delphi process. The Delphi process will allow us to validate and potentially expand upon our initial findings, ensuring no crucial reporting elements are missed regardless of their source.

- Provide more details on how mixed methods experts will be identified, both in terms of the literature to be searched and the criteria for selecting experts.

We have expanded our description of expert identification to detail both our search strategy and selection criteria. We added more details into the text. “Expert identification will involve a multi-faceted approach to ensure comprehensive representation across disciplines. For this study, experts are defined as individuals with demonstrated knowledge and skills in mixed methods research. The selection criteria is defined in Table 1. We will begin by extracting author information from our scoping review of mixed methods literature. This will be supplemented by a targeted literature search in Scopus and Web of Science using terms such as 'mixed methods research' and 'mixed methodology' to identify authors who have published extensively in this field. We will focus on authors who have published multiple mixed methods studies or methodological papers about mixed methods research. Authors' institutional profiles and Google Scholar profiles will also be reviewed to confirm their ongoing engagement with mixed methods research. The lead author will compile an initial list of experts, categorized by research discipline, which will be reviewed and supplemented by the full research team to ensure comprehensive representation. The experts' biographies and publication records will be consulted to verify their research design expertise, including their research interests, courses taught, and scientific publications. Furthermore, we will contact the Mixed Methods International Research Association and other relevant organizations to nominate experts based on pre-determined selection criteria (Table 1). To address disciplinary differences, we will strive for a balanced representation of experts across various fields, including but not limited to psychology, sociology, health sciences, and education.”

- Consider reviewer 1’s suggestions regarding measurement tools, criteria for agreement, and other methodological issues.

Upon the discussion with the team we came to agree with the reviewer and adjusted our questionnaire to address the reviewer’s comments. We thank the reviewer for these methodological suggestions and have made several specific adjustments to strengthen our measurement approach. We have revised our consensus criteria and measurement tools as follows: We now use a 10-point Likert scale instead of the originally proposed 5-point scale, allowing for more nuanced expert assessment of items. We have also modified our consensus thresholds, using ≥85% agreement in the first round (defined as scores 7-10 on the Likert scale), followed by a 75% threshold in subsequent rounds. Additionally, we have added a separate 3-point scale for rating item clarity (1- completely unclear, 2- somewhat clear, and 3 – completely clear) to ensure both item retention and clarity are systematically assessed. Each item is also followed by an open-text field allowing experts to provide detailed feedback and justification for their ratings.

- Clarify how the 10 articles for the pilot will be selected.

We appreciate this comment and have refined our pilot testing approach. We have increased the number of articles from 10 to 15 to ensure broader representation across disciplines, and have clarified our selection strategy. The pilot articles will be selected through a structured process: First, we will conduct a search in Scopus and PsycINFO to identify mixed methods studies published in the last three years. To ensure disciplinary diversity, we will stratify our selection to include articles from four major disciplines (psychology, sociology, health sciences, and education), aiming for 3-4 articles from each field where feasible. We will use convenience sampling within each disciplinary stratum, selecting articles that demonstrate varied approaches to mixed methods research. This balanced representation across disciplines will allow us to assess the checklist's applicability and usefulness across different research contexts and methodological traditions. The same set of articles will be reviewed by all members of the pilot team to enable consistent assessment of the checklist's reliability and usability.

- Respond to any other issues raised by the reviewers.

We addressed all comments raised by the reviewers.

- There are several typographical and grammatical errors throughout the paper. These need to be corrected: e.g. lines 64-65, 101-102.
We thank the editor again and we reviewed the manuscripts for typographical and grammatical errors.

----

Reviewer #1: A this point, I still have a number of comments, most of which are rather small.

On lines 117-118, the authors state that their tool will address the “philosophical underpinnings of mixed methods,” and on 178-179 they state the need to prompt “researchers to report their philosophical or paradigmatic stance.” But isn’t this something that the experts should decide, rather than having the authors make prior assumptions about the centrality of this topic?

Thank you for your comment regarding the philosophical underpinnings of mixed methods research. We agree that the importance of reporting philosophical and paradigmatic stances should ultimately be determined by the expert consensus process rather than by prior assumptions from the authors. The literature review and scoping review we conducted suggest that experts may consider these aspects important to report, but you rightly point out that this should be confirmed through the Delphi process. To address this, we have amended the manuscript to clarify that the expert panel will be responsible for deciding which elements, including philosophical and paradigmatic considerations, are essential to include in the CORMIX reporting guideline. The revised wording acknowledges the key role of expert consensus in determining the final checklist items, while still noting that our preliminary work indicates these aspects could be relevant for discussion. We appreciate you raising this important point and prompting us to refine our language around the reporting philosophical stances. Ultimately, the expert consensus process will be the arbiter of which items are included in CORMIX.

On line 200 they mention searching in the Journal of Mixed Methods Research, and I would add the International Journal of Multiple Research Approaches.

Thank you for suggesting the addition of the International Journal of Multiple Research Approaches to our search strategy. We appreciate you bringing this relevant journal to our attention. Since receiving your feedback, we have completed a separate search of this journal to ensure comprehensive coverage of the literature. It's important to note that the International Journal of Multiple Research Approaches is indexed in databases we had already searched, including PubMed and ProQuest. Therefore, our original search strategy likely captured articles from this journal. However, to be explicit and thorough, we have now included the International Journal of Multiple Research Approaches in the list of journals specifically searched, as detailed in the revised manuscript.

On line 267 the authors state that they will conduct “a targeted literature search to identify
additional mixed methods experts.” I find this to be too vague, since it doesn’t say anything about which literature will be searched or what criteria will be used for selection. I have personally conducted several citation searches that being with “mixed method*” and the results are so widely scattered that they would be useless for locating experts in the field.

Thank you for raising this important point. To address this, we have expanded upon the expert identification process in the revised manuscript. The amended text now reads: "This will be supplemented by a targeted literature search in Scopus and Web of Science using terms such as 'mixed methods research' and 'mixed methodology' to identify authors who have published extensively in this field. We will focus on authors who have published multiple mixed methods studies or methodological papers about mixed methods research. Authors' institutional profiles and Google Scholar profiles will also be reviewed to confirm their ongoing engagement with mixed methods research."

On lines 272-273 the authors say they will use snowball sampling to expand their list of experts. This is a useful strategy, but snowball sampling is highly sensitive to the initial set of “seeds” that are used. So, this reinforces the point that I just made above about the importance of an explicit strategy for selecting the first set of experts.

Thank you for emphasizing the importance of the initial set of experts when using snowball sampling to expand our expert panel. We recognize that the quality and representativeness of the experts identified through snowball sampling are highly dependent on the starting point. To ensure a robust initial set of experts, we have further detailed our selection strategy in the revised manuscript. As mentioned in the previous response, we will begin by conducting a comprehensive literature search in Scopus and Web of Science using key terms such as 'mixed methods research' and 'mixed methodology' to identify authors with extensive publications in the field. We will prioritize authors who have published multiple empirical mixed methods studies or methodological papers, and we will verify their ongoing engagement with mixed methods research through their institutional profiles and Google Scholar records. In addition to this literature-based approach, we will leverage our connections with the Mixed Methods International Research Association (MMIRA) to nominate experts who meet our predefined selection criteria. MMIRA is a leading professional organization dedicated to the advancement of mixed methods research, and its membership includes many highly qualified and experienced mixed methods researchers from various disciplines. By starting with experts recommended by MMIRA, we can be confident that our initial set of experts will be knowledgeable and well-respected in the field. From this carefully selected initial group of experts, we will then employ snowball sampling to expand our panel. The experts identified through MMIRA and our literature search will be asked to nominate additional experts who meet our inclusion criteria. This approach allows us to leverage the professional networks of established mixed methods researchers while maintaining the rigorous standards set forth in our selection process. Thank you again for highlighting the sensitivity of snowball sampling to the initial set of experts, as it has prompted us to further clarify and strengthen our expert selection methodology in the manuscript.

Line 303 states that the items will be evaluated “using a 5-point Likert scale from highly relevant to non-relevant.” In my experience, this will lead to a highly skewed set of results, because very few of the items will be “non-relevant” after the authors’ intense screening process. I suggest that the scale run from “Minor Relevance” to “Highest Relevance” and that the authors use a 10-point rating scale. The usual argument for using 5-point and shorter scales is that it reduces respondent “burden,” but that is not a major issue when working with a highly educated panel who have all volunteered to engage in a rating process.

We appreciate you sharing your experience and suggesting a modification to improve the discriminatory power of the scale and the quality of the data collected. After careful consideration and consultation among the research team, we have decided to adopt your recommendation. . As such we have now amended the scale to use a 10 point likert scale instead of 5 point. Furthermore, we have refined the focus of the rating scale to assess the suitability of each item for inclusion in the final reporting checklist. The revised instructions will ask experts to rate the extent to which they agree that each item should be retained in the final CORMIX checklist.

On line 303 the rating scale is described as “highly relevant to non-relevant,” but on lines 320-321 it is described as “Strongly disagree” to “Strongly Agree.” Again, no one is like to strongly disagree with the relevance of these items. If items “pile up” at the top of the rating scale, then al most all of them will meet the authors’ criteria for consensus, which would lead to a very lengthy instrument. As above, I would recommend a 10-point scale, and then use scores of either 7-10 or 8-10 as a cut-off for consensus.

Again, we thank the reviewer for their comment. The rating scale for evaluating the suitability of items for inclusion in the final CORMIX checklist has been modified to a 10-point Likert scale. This change was made in response to the reviewer's thoughtful suggestion and aims to enhance the discriminatory power of the scale and the quality of the data collected during the Delphi process. To determine consensus, the authors will now consider items with scores of 7 to 10 as having achieved consensus for inclusion in the checklist. Manuscript modifications were completed accordingly.

Line 323 I disagree about whether there is agreement on the figure 85% for consensus. One common joke among Delphi researchers is that “there is no consensus about consensus.” I personally would recommend that 85% be set as an aspirational goal, but that 75% after the second round be considered adequate.
Again, we thank the reviewer for sharing their expertise. You are correct that there is no universally agreed-upon figure for defining consensus, and the choice of threshold can vary depending on the study's context and objectives. After careful consideration and further consultation with experts in Delphi methodology, we have decided to amend our approach to defining consensus. We agree that setting 85% as an aspirational goal for the first round, while considering 75% as adequate for subsequent rounds, is a reasonable and pragmatic approach. In the revised manuscript, we now state that consensus will be defined as follows: “In the first round, items that receive agreement (scores of 7-10 on the 10-point Likert scale) from 85% or more of the experts will be considered to have reached consensus for inclusion in the CORMIX checklist. For subsequent rounds, the consensus threshold will be set at 75%.”

On line 345, I have found that one of the most useful aspects of the comments made by the experts is to adjust the wording of items. Will this be possible?
yes, experts will be asked to rate each item on a 3-point Likert scale on clarity (1 completely unclear, somewhat clear and completely clear) they will also be asked to provide comments in an open text format related to the clarity and to recommend any other modifications. We have made this explicit in the manuscript.

In the section on lines 347 to 367 it is unclear whether the 10 people are each going to rate all 10 articles. If so, I would prefer to have a sub-sample of the panel rate a larger number of articles. You would have the same number of ratings if five people each rated 20 articles. The reason for this recommendation is that variability among the articles is at least as important as variability among the raters.
Thank you for raising this important point about the piloting process for the CORMIX checklist. In our initial submission, we had proposed having fewer than 10 people in the piloting team. However, based on the valuable feedback from the reviewers, we increased the number to 10 to enhance the representativeness of the pilot testing process. To strike a balance between the workload for the piloting team and the need for variability among the articles, we have now opted to have each of the 10 team members independently apply the checklist to a set of 15 sampled mixed methods studies from different fields. It is important to note that CORMIX is a reporting guideline rather than a quality assessment tool. As such, the primary focus of the piloting process is to evaluate the clarity, comprehensiveness, and ease of use of the checklist items in the context of reporting mixed methods research. The goal is to ensure that the checklist can be effectively applied by researchers, reviewers, and editors to improve the transparency and completeness of mixed methods study reports.

We have amended the relevant sentence in the manuscript to reflect this change: "This piloting team will independently apply the checklist to the same set of 15 sampled mixed methods studies across different fields."

Line 360 a random sample of “mixed method studies published in the last three years” will have a population of about 3,000 articles. How will the authors “stratify” such a large population to get just 10 (or 20) matching articles? I also worry that relying on PubMed for half the sample frame will lead to an over-emphasize health-related topics. What about substituting Scopus instead?

We agree that relying solely on PubMed for half of the sample frame could lead to an overemphasis on health-related topics. To address this concern, we have decided to substitute PubMed with Scopus, as you suggested. Regarding the stratification of the large population of mixed methods studies published in the last three years, we will employ a two-step approach. First, we will conduct a search in Scopus and PsycINFO using relevant keywords to identify mixed methods studies published within the specified timeframe. From the resulting pool of articles, we will then stratify the sample by major focus areas, such as psychology, sociology, health sciences, and education. This stratification will help ensure that the selected articles represent a diverse range of disciplines in which mixed methods research is commonly applied. Once the articles have been stratified by focus area, we will randomly select a predetermined number of articles from each stratum to achieve a total sample size of 15 articles for pilot testing. By using this stratified random sampling approach, we aim to obtain a representative sample of mixed methods studies that spans different disciplines while maintaining the feasibility of the piloting process. We have updated the relevant section of the manuscript to reflect these changes: "The fifteen articles will be identified through a search in Scopus and PsycINFO for mixed method studies published in the last three years. To ensure balanced representation, articles will be stratified by major disciplines (psychology, sociology, health sciences, and education), and randomly selected from each stratum to achieve a total sample size of 15."

Reviewer #2: The authors have addressed the comments of the reviewers. Yet, there are still some remaining issues. The main comment is about the timeline suggested. The authors are planning a search period of the literature until September 2024 (line 225), a recruitment period of experts in September 2024 (line 298), and start the Delphi phase in October 2024 (line 299). Considering that the results of the review will be used to develop the Delphi questionnaire, it does not seem feasible to complete all the steps of the review, and develop and validate the questionnaire within one month. If the authors have already completed the review and questionnaire, they should include the actual date of the search, and state that the review is completed in the protocol.

We thank the reviewer for their valuable comments. We have completed several key phases of the project. The scoping review has been completed, yielding 50 relevant articles from an initial screening of 4,978 articles. From these articles, we have completed data extraction and synthesis, resulting in a preliminary list of items for the Delphi phase (provided in supplementary file). Additionally, the questionnaire for the Delphi phase has been developed and validated by the research team. Expert recruitment is currently ongoing, and based on our progress and existing commitments from experts, we anticipate completing expert recruitment by end of November, with the first Delphi round beginning in December 2024, followed by subsequent rounds through February-March 2025. This timeline is feasible given the substantial groundwork already completed and allows adequate time for expert engagement between rounds. We have amended the manuscript to reflect this timeframe.

Other comments are listed below:
• Line 106: replace “GRAMMAS” by “GRAMMS”.

Comment addressed
• Line 166: Spell out “MMAT” since the first time it appears in the text.

Comment addressed

• Lines 182-184: The authors mentioned “The MMR-RHS checklist is tailored for rehabilitation and health sciences. While it may be adaptable to other fields, it might not fully capture the nuances of mixed methods research in other disciplines”. To help readers understand, the authors should add an example of nuances of mixed methods not captured in the MMR-RHS.

We thank the reviewer for the comment. We amended the paragraph to add an example. The text now reads “The MMR-RHS checklist, while well-designed for rehabilitation and health sciences, may not fully capture the nuances of mixed methods research across different disciplines. For instance, in social sciences, philosophical stances (e.g., pragmatism, constructivism, transformative paradigms) often fundamentally guide research design, yet the MMR-RHS doesn't explicitly prompt reporting of these considerations. This limitation is also seen in other tools like ASSESS, despite philosophical and paradigmatic considerations being widely discussed in mixed methods literature. Given these gaps in existing reporting frameworks, our study will use expert consensus to evaluate the role and importance of reporting such considerations in mixed methods research.”

• Line 219: The authors mentioned using free keywords and controlled vocabulary (MeSH and EMTREE). Yet, it is not clear what controlled vocabulary could be used for this topic. As mentioned previously, if the review is completed, the authors could add the actual search strategy used.

Upon completion of the review, we opted for the use of free text as it provided more hits while the MeSH and EMTREE were extremely limited. We have now completed this process and provided the search strategy used in the supplementary file.

• Lines 238-241: “A two-stage screening process will be employed, first: initial screening will focus on mixed methods-specific guidelines. Second, quantitative and qualitative checklists will be reviewed selectively, prioritizing those frequently cited in mixed methods literature or those that have significantly influenced reporting practices”. This description of the screening process is different from what is usually found in reviews (i.e., first, screen titles and abstracts and then full-text papers). The process suggested will involve first identifying guidelines and then checklists. This could be done at the same time (i.e., identifying guidelines and checklist from titles/abstracts and then from full-text papers).

We appreciate the reviewer's comment about the screening process. The screening process followed standard systematic review procedures: first, titles and abstracts were screened by two independent researchers using Rayyan, followed by full-text screening of potentially relevant articles. Our search identified 13,439 articles, which after duplicate removal resulted in 4,978 articles for title/abstract screening. Full-text screening was completed for 121 articles, with 50 articles ultimately included. We provide clarification in the manuscript accordingly.

• Lines 241-243: “We will limit our in-depth review to the top 10 most relevant checklists for each methodology (quantitative and qualitative) based on citations.” It is not clear why the authors will limit to the top 10 checklists. Also, using number of citations to judge which ones are the “most relevant” can also be misleading. For example, more recent checklists are likely to be less cited but can be more relevant compared to a checklist developed in the 80s.

We appreciate this thoughtful observation about the potential limitations of using citation counts and restricting review to top 10 checklists. Our primary focus was on mixed methods-specific reporting guidance, with qualitative and quantitative checklists serving as supplementary sources to ensure comprehensive item identification. The selection of frequently cited checklists was not meant to be exhaustive but rather to complement our main findings from the scoping review specific to mixed methods literature. We believe that any potential limitations from this approach would be mitigated through our comprehensive methodology. Firstly, our scoping review captured reporting items from mixed methods literature regardless of their source or citation count, ensuring we didn't miss important elements. We also believe that the expert panel participating in our Delphi process would bring in extensive experience with reporting guidelines across methodologies. Their expertise helps ensure that crucial reporting elements are not overlooked, regardless of their origin in newer or older checklists. The Delphi process will include opportunities for experts to suggest additional items that may not have emerged from the literature review, providing another safeguard against missing important reporting criteria.
• Line 282: “This will be supplemented by a targeted literature search to identify additional mixed methods experts.” The authors should describe how they will process (e.g., where will they find the literature, how will they select the literature, etc.).

We have described the process in more detail “This will be supplemented by a targeted literature search in Scopus and Web of Science using terms such as 'mixed methods research' and 'mixed methodology' to identify authors who have published extensively in this field. We will focus on authors who have published multiple mixed methods studies or methodological papers about mixed methods research. Authors' institutional profiles and Google Scholar profiles will also be reviewed to confirm their ongoing engagement with mixed methods research”

• Lines 381-388: “These studies will be systematically selected to represent a range of disciplines and reporting quality. The ten articles will be identified through a systematic search in PubMed and PsycINFO for mixed method studies published in the last three years. Stratified random sampling of the ten articles will be conducted to ensure representation across disciplines.” How will the authors assess the reporting quality of the 10 papers? Also, considering the latter sentence (random sampling), it seems that the authors will first identify all published mixed methods studies from the past 3 years indexed in PubMed and PsycINFO. Then, they will appraise the reporting quality of all identified mixed methods studies and classify them into disciplines. They will then randomly select 10 based on disciplines and reporting quality. This seems to be very time consuming. For example, only in Medline, there are more than 7000 papers published since 2022 that have used mixed methods in their titles.

Thank you for highlighting this issue. You raise important points about the feasibility and purpose of our sampling strategy. We have revised our approach to better align with our primary objective of testing the checklist's usability rather than assessing reporting quality of published papers. We agree that our original description implied an unfeasible and unnecessarily complex process. We have modified our sampling approach and clarified our methodology as follows: (1) We have increased the sample to 15 articles (rather than 10) to ensure better disciplinary representation, (2) We will use convenience sampling instead of systematic random sampling, (3) Articles will be identified through focused searches in Scopus and PsycINFO from the last three years, (4) We will select 3-4 articles from each of four major disciplines (psychology, sociology, health sciences, and education) to ensure balanced representation. We have removed references to assessing reporting quality as this is not our objective. The purpose of this pilot phase is to test whether the checklist is clear, applicable, and user-friendly across different disciplines, not to evaluate the quality of existing literature.

The revised text in the manuscript now reads: "This piloting team will independently apply the checklist to the same set of 15 sampled mixed methods studies across different fields. These studies will be selected through convenience sampling to include studies from various disciplines. The fifteen articles will be identified through a search in Scopus and PsycINFO for mixed method studies published in the last three years. To ensure balanced representation, articles will be stratified by major disciplines (psychology, sociology, health sciences, and education), aiming for approximate balance across fields where feasible."

• Reference #1 refers to two different books by Creswell (Designing and conducting mixed method research VS Research design: Qualitative, quantitative and mixed methods approaches). The authors should check which want they wanted to cite.

We apologize for this error we corrected the reference.

• Reference #5 is incomplete. Please check.

Comment addressed

• Figure 1: There is a missing word at the end of step 6.

Comment addressed
• The authors should avoid using contractions in the manuscript (e.g., there’s (line 81), It’s (line 118), …).

Manuscript was reviewed and all contractions were removed.
