# Supplementary material for: Protocol for developing a Consolidated Checklist for Reporting Mixed Methods Research (CORMIX) using modified Delphi
Source: PLoS One. 2025 May 6;20(5):e0321587. doi: 10.1371/journal.pone.0321587 (PMC12054909; doi:10.1371/journal.pone.0321587)
Supplement: Attachment — Submitted filename: Response to Reviewers R3.docx [file pone.0321587.s006.docx]

Jan 19^th^ , 2025

The Editor-in-Chief

PLOS ONE

Dear Dr. Fàbregues

**Revision of Manuscript**

**PONE-D-24-20917R2**

**Protocol for Developing a COnsolidated Checklist for Reporting MIXed-Methods Research (CORMIX) Using Modified Delphi.**

Thank you for your additional feedback on our manuscript. We appreciate the opportunity to address the points raised by the editor. We wish to highlight that we only received the comments shared by the editor without new comments from the reviewers. We are positive that the comments helped in enhancing the quality and scientific merit of the paper.

Below are separate pages, detailing our responses to the comments of the journal requirements and editor.

**Please note that changes are made using “track changes” tool within the revised manuscript.**

Thank you to the editor for the time spent on this manuscript. We look forward to receiving your kind response.

Sincerely yours,

**The Corresponding Author**

**All in all, we thank you for your great review which we believe would add great value to the adjusted manuscript.**

**Detailed Responses to the Comments of the Journal Requirements and Editor**

Journal Requirements:

Please review your reference list to ensure that it is complete and correct. If you have cited papers that have been retracted, please include the rationale for doing so in the manuscript text, or remove these references and replace them with relevant current references. Any changes to the reference list should be mentioned in the rebuttal letter that accompanies your revised manuscript. If you need to cite a retracted article, indicate the article’s retracted status in the References list and also include a citation and full reference for the retraction notice.

We have reviewed each reference and manually adjusted any inconsistencies. No retracted paper was cited in this research. All changes were made using track changes. These are the changes in brief:

Journal names were all abbreviated. Edition and volume were added in few missing references. Standardized formatting for references, in terms of punctuation and doi links.

Additional Editor Comments:

While the authors have addressed most of the reviewers’ comments, the reviewers note that a few minor issues still need to be addressed. These issues, with which I agree, include:

- Clarify how all the steps of the review will be completed in the short period of time mentioned between the date of the search and the start of data collection.

We confirm that several key phases have already been completed. (1) Scoping review (completed September 2024): 50 relevant articles identified from 4,978 screened articles. (2) Questionnaire development and validation (completed October 2024). (3) Expert recruitment (began December 2024, currently ongoing)

To better illustrate this progress, we have added Figure 3 showing completed steps and timeline under a new section “Completed steps" in the manuscript

- Correct several typos and add some examples, as suggested by the reviewers.

We conducted a thorough review of the manuscript and corrected all typos. In the previous review, we added an example related to philosophical stances in mixed methods research “For instance, in social sciences, philosophical stances (e.g., pragmatism, constructivism, transformative paradigms) often fundamentally guide research design, yet the MMR-RHS doesn't explicitly prompt reporting of these considerations.”

- Clarify and add additional information about the methods employed (especially regarding the search process for identifying experts) and their limitations.

Based on the previous review, we have expanded our description of expert identification methods to include detailed selection criteria in Table 1, systematic literature searches in Scopus and Web of Science, professional network outreach through MMIRA, snowball sampling from identified experts. We also added a paragraph acknowledging the limitations of our approach. “We acknowledge several limitations in our expert identification strategy. First, reliance on academic publications may underrepresent practitioners and experts who primarily contribute through other channels. Second, English-language dominance in major databases could limit identification of experts from non-English speaking regions. Third, citation metrics and publication counts, while useful indicators, may not fully capture expertise, particularly for emerging scholars or those focusing on specific disciplinary applications of mixed methods. To mitigate these limitations, we will actively seek recommendations from professional organizations and use snowball sampling to identify experts who might be missed through traditional academic metrics. We will also make specific efforts to include experts from diverse geographical regions and academic traditions.”

- Correct several problems in the list of references.

We have reviewed and updated all references.

- In the response to the reviewers letter, provide a justification for procedures and steps that were performed in a different way than suggested by the reviewers

We thank the editor for their comment. We have addressed and agreed with almost all comments raised by the reviewers in the previous review rounds. To summarize these were the changes and their justifications:

The reviewer suggested using a 10-point Likert scale instead of our originally proposed 5-point scale. We agreed with this suggestion and implemented it, as a 10-point scale offers better discriminatory power when working with expert panels. Additionally, we modified our consensus thresholds to ≥85% agreement (scores 7-10) and subsequent rounds: ≥75% agreement. This modification allows for more nuanced expert assessment while maintaining rigorous consensus standards.

We modified our database selection from PubMed to Scopus and PsycINFO as suggested in previous rounds of review as it provides broader disciplinary coverage, reduces potential bias toward health sciences and better captures social science and education research allowing more comprehensive identification of mixed methods studies.

While maintaining the suggested snowball sampling approach, we expanded our expert identification strategy to include systematic database searches in Scopus and Web of Science, engagement with professional organizations (MMIRA), and review of institutional profiles and Google Scholar metrics. This comprehensive approach helps ensure a diverse, qualified expert panel while addressing potential limitations of any single identification method.

While a reviewer suggested having fewer people rate more articles for the pilot of the tool, we opted for a different approach: Increased the number of articles from 10 to 15 and maintained the full pilot team of 10 members rating all articles. This approach provides broader disciplinary representation, and greater reliability in assessing the checklist's usability through better assessment of inter-rater consistency.

We believe we have taken all reviewers comments raised in previous rounds into consideration. These modifications from the original suggestions were made to enhance the scientific rigor of our study while maintaining feasibility and ensuring comprehensive representation across disciplines. Each decision was made after careful consideration of the reviewers' feedback and practical constraints of the study.
